# A Resume of Things Concerning the Medical Officers’ Training Camp at Fort Benjamin Harrison

**Published:** 1917-09

**Authors:** Samuel E. Earp

**Affiliations:** Indianapolis; Editor Indianapolis Medical Journal


### A Resume of Things Concerning the Medical Officers' Training Camp at Fort Benjamin Harrison.

By SAMUEL E. EARP, M. D., Indianapolis,

Editor Indianapolis Medical Journal.

At the gates of Indianapolis is situated Fort Benjamin Harrison, one of the training camps for officers of the United States Army. There is a post hospital of one hundred beds, and all else that goes with such an institution, including accessory camps for the care of those affected with contagious diseases. There is an infirmary sick call at 4 p. m. each day those sick are transferred to the hospital. The barracks will accommodate nearly a thousand medical officers and is lighted by electricity. The mess is unusually good, but in this, like some other things, a few cents extra will take to the extreme, which is unnecessary. Lectures and quizzes are conducted under trees, and blackboard exercises are in evidence, and thus the instructors find out what has been accomplished during study and drill hours.

Recreation consists of all kinds of athletic and other games. Entertainments are of the best. The governors of several states have delivered lectures, and Senator James Watson and a number of others of equal rank in Washington, have done their bit. None excel instruction, advice, and words of truth that are given by Brigadier-General Edwin F. Glenn. Enthusiasm is at high pitch, co-operation is of the best and discipline is of a superior quality.

At the post there are now 13,000 men. Through the courtesy of Major Percy M. Ashburn, I obtained information which is authentic. The morning that I visited the post, July 23, in the interest of the Buffalo Medical Journal, the written morning report shown me gave the number of men in the medical training camp as 2842. There are four field hospitals of 80 men, four ambulance companies of 150 men, one evacuation hospital of 180 men, 20 regimental attachments and 33 men in sanitary squad. In addition there is one Indiana Field Hospital of the Indiana National Guard, one ambulance company of the Indiana National Guard, and 400 enlisted men from different states.

The brain and body work of the student officers commences at 7 a. m. and continues until 5 p. m. The work is divided into three parts: 1st month experience of enlisted men and learning duties in medical deportment; 2nd month, officers' duties, and 3rd month includes both 1st and 2nd.

The best men adapted to particular service are recommended for advancement by Major Ashburn, and the appointment is made at Washington.

There is a parade and review each day but the student medical officers have had but one review, and that was when Governor Cox of Ohio visited the post.

Those who have left the camp had some physical disability and none for inefficiency. This is surely creditable to a superlative degree. In line of duty 100 have gone elsewhere, 75 abroad, (and we know what that means), and 35 assigned to duty as instructors at other camps. These facts until now were unpublished.

Incidentally, it might be said that the student medical officers have no gun practice but they do have hikes of six miles a day, two or three hours of drill, and special drill in ambulance duties and other medical work. The men get the best food and water. Two new filter-beds were put in this week and there are deep wells that reach below the blue glacial clay, which means the second stream of water. There are no 30 feet surface wells.

Saloons and houses of prostitution are not permitted nearer than five miles from the camp. There is one exception, if a saloon man had his license before the adoption of this rule it would not exclude him if the saloon was located one-half mile from the post, but no liquor is allowed at the post, no lewd women, and soldiers cannot be given liquor when in uniform during a visit to the city when off duty. Anyone giving liquor to soldiers is subject to arrest and imprisonment upon conviction.

There have been no cases of alcoholism at the post and at this writing no known case of venereal disease. This undoubtedly is a creditable record.

A word about court-martial. A captain of the Spanish-American War, Dr. Eugene Buehler, and business manager of the Indianapolis Medical Journal, and who accompanied me to the post, said: "We had a case of court-martial almost every day." Major Ashburn who heard the remark replied: "This post opened June 1st and we have had two trials only, both for absence without permission."

Many of the student officers are college students or graduates, and those who compose this camp are of the highest quality and the best character.

The officers of the medical training camp are: Major Percy M. Ashburn, chief officer; Major J. R. Shook, Major G. H. Scott and Majors Morse and Powell. Those of the rank of captain are: Captains Snyder, MacCormack, Druly, Bastian, Creighton and Turnbull.

The men of the camp co-operate perfectly with these officers and they are held in the highest esteem. Fort Benjamin Harrison is a great camp for good and those who go forth from it will be an integral part in the successful termination of the world's war.

634 Occidental Building, Indianapolis, Ind.

P. S. The editor acknowledges the great courtesy of his colleague in preparing this article. Various questions have been asked by those expecting to be ordered to this camp. Dr. Earp answers them as follows: Some men bring a trunk and suit case and dispose of such baggage and containers as are not needed. The general order during war is against the use of civilian clothing at any time. Some have brought autos and get time to use them when on leave of absence, especially parts of Saturdays and Sundays. Those who have contemplated traveling to camp by auto and selling it on leaving, must expect to do so at considerable loss. General orders are that medical reserve officers should equip themselves with uniform, cot, bedding, etc., in advance, as stated in bulletins and lists issued by the Surgeon General, but as compliance with this order is in some cases impracticable, quite a few wait till they reach Indianapolis before securing equipment and some until they actually arrive at camp. Major Ashburn's remark about court martials indicates that the omission is not considered serious, although all should equip themselves so far as possible.

---

**Anatomic Localization of Projectiles in the Posterior Aspect of the Heart.** René Le Fort, Le Progres Med., June 2, has had an experience with 25 cases. Radiology gives valuable but often incomplete assistance and the surgeon must expose sufficiently to complete the diagnosis. In one case, a ball had remained in the posterior wall of the right ventricle for 30 months. Complete cardiac symphysis prevented the use of the posterior wall of the heart up to the esophagus, separate adhesions from the rear forward, penetrate the cardiac wall and disengage the firmly imbedded ball. In another case, the extreme movability of a shrapnel bullet behind the pericardium prevented its seizure. The pericardium was then incised in front of the phrenic nerve and the ball was slid up to the oesophagus and held in place by the fingers while it was extracted through a second button-hole opening. In seven cases, one with the ball lodged in the posterior wall of the heart, six just behind it, the mortality was zero.

